# Incidence of childhood type 1 diabetes mellitus in Yamanashi Prefecture, Japan, 1986‐2018

**DOI:** 10.1002/edm2.214

**Published:** 2020-12-14

**Authors:** Tomohiro Saito, Koji Kobayashi, Kisho Kobayashi, Mie Mochizuki, Hideaki Yagasaki, Koichi Makino, Hiromune Narusawa, Daisuke Watanabe, Yumiko Mitsui, Kazumasa Sato, Tomoaki Sano, Masanori Ohta, Hiroshi Yokomichi, Shin Amemiya

**Affiliations:** ^1^ Department of Pediatrics Faculty of Medicine Graduate School of Medicine University of Yamanashi Yamanashi Japan; ^2^ Department of Pediatrics Yamanashi Prefectural Hospital Yamanashi Japan; ^3^ Department of Pediatrics Yamanashi Kosei Hospital Yamanashi Japan; ^4^ Kobanyashi Kids’ Clinic Yamanashi Japan; ^5^ Department of Pediatrics Kyonan Medical Center Fujikawa Hospital Yamanashi Japan; ^6^ Department of Pediatrics Fujiyoshida Municipal Hospital Yamanashi Japan; ^7^ Ryuoh Mitsui Clinic Yamanashi Japan; ^8^ Department of Pediatrics Yamanashi Red Cross Hospital Yamanashi Japan; ^9^ Department of Pediatrics Tsuru Municipal General Hospital Yamanashi Japan; ^10^ Department of Health Sciences Faculty of Medicine Graduate School of Medicine University of Yamanashi Yamanashi Japan; ^11^ Department of Pediatrics Faculty of Medicine Saitama Medical University Saitama Japan

**Keywords:** age of onset, diabetes mellitus type 1, incidence, Japan, paediatrics

## Abstract

**Introduction:**

Several studies have examined the incidence of childhood T1DM in Japan from the 1970s onwards, but none have been long‐term studies using registration data. We estimate the incidence of childhood type 1 diabetes mellitus (T1DM) from 1986 to 2018 in Yamanashi Prefecture, Japan.

**Methods:**

We began a population‐based, long‐term study of childhood T1DM in 1986 involving every hospital paediatrics department in Yamanashi Prefecture. In the Prefecture, every child newly diagnosed with T1DM is referred to a hospital, and therefore, almost 100% of new patients aged <15 years are registered. We calculated the incidence of T1DM among children aged <15 years from 1986 to 2018. All cases met the Japan Diabetes Society diagnostic criteria and were tested for T1DM‐related autoantibodies whenever possible.

**Results:**

Ninety‐nine patients (44 boys and 55 girls) were newly diagnosed with T1DM. The annual incidence among 5‐ to 9‐year‐olds increased by 5.35% over the study period (95% confidence interval 2.34%‐8.35%, *p* = .0005), and there was a trend towards increasing 3‐year incidence (15.52% increase, *p* = .0516). There were also trends towards increasing annual and 3‐year incidence among 0‐ to 14‐year‐olds. However, there were no changes over time in annual or 3‐year incidence in the 0–4 year or 10–14 year age groups.

**Conclusions:**

The incidence of T1DM in Yamanashi Prefecture increased among children aged 0‐14 years over the study period, with the most significant increase occurring among 5‐ to 9‐year‐olds. These data suggest that the number of children aged <15 years with T1DM is gradually increasing in one of the local prefectures in Japan, Yamanashi Prefecture and that the age of onset is decreasing.

## Graphical abstract

The 3‐year incidences of type 1 diabetes in each age group between 1986 and 2018 in Yamanashi, Japan. The rate of increase in 5‐ to 9‐year‐old children was 15.52% (*p* = .0516, *n* = 22, Poisson regression test).

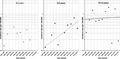

## 1 INTRODUCTION

Matsuura et al reported that the incidence of type 1 diabetes mellitus (T1DM) in children aged <15 years in Japan increased from 0.98/100 000 person‐years from 1973 to 1977 to 2.53/100 000 person‐years from 1988 to 1992.^1^ However, Kawasaki et al reported T1DM incidences of 2.00/100 000 person‐years from 1993 to 1996 and 2.37/100 000 person‐years from 1998 to 2001, concluding that there was no evidence of increased T1DM incidence among children aged <15 years between 1988 and 2001 in Japan.^2^ Many chronic diseases, such as T1DM, Graves' disease, systemic lupus erythematosus and acute lymphocytic leukaemia, are defined as ‘Specific Chronic Pediatric Diseases’ in Article 6‐2 of the Child Welfare Act of Japan. Children with these conditions can receive financial support to defray medical costs via registration in a government‐funded medical aid project. Recently, Onda et al estimated the incidence of childhood T1DM in Japan as 2.25/100 000 person‐years from 2005 to 2010 based on the number of recipients of healthcare subsidies for ‘Specific Chronic Pediatric Diseases’. The authors compared their data with those from previous studies in Japan and concluded that the incidence of childhood T1DM in Japan had not changed between 1998‐2001 and 2005‐2010.^3^ Thus, from the 1970s onwards, several studies have examined the incidence of childhood T1DM in Japan. However, none have been long‐term studies using registration data.

It has been reported that the incidence of T1DM among children aged <15 years is increasing in various regions globally. The European Community Concerted Action Programme in Diabetes (EURODIAB) reported that the incidence of T1DM among children aged <15 years in Europe increased between 1989 and 2003, and if this trend continued, that incidence would double between 2005 and 2020.^4^ The incidence of T1DM in this age group from 2011 to 2013 in Kuwait was 2.3 times higher than that from 1995 to 1997.^5^ In Croatia, there was a 5.87% increase in the incidence of T1DM among children aged <15 years between 2004 and 2012 (*p* < .001),^6^ and the incidences of T1DM among Jewish and Arabic children aged <18 years in Israel increased by 3.6% and 4.0%, respectively, between 1997 and 2010.^7^ In Bauru, Brazil, the incidence of T1DM among children aged <15 years increased at a rate of 3.1% per year, resulting in an overall increase of 2.5‐fold between 1986 and 2015.^8^ In Korea, the incidence of T1DM in this age group increased from 1.36/100 000 person‐years from 1995 to 2000 to 3.19/100 000 person‐years from 2012 to 2014.^9^ In Uzbekistan, the incidence of T1DM among children aged <15 years increased from 1.5/100 000 person‐years in 1998 to 3.8/100 000 person‐years in 2014, with an average annual rate of increase of 5.6% (*p* < .001).^10^

To assess temporal trends in the incidence of T1DM among children in Japan, we registered all children newly diagnosed with T1DM in Yamanashi Prefecture from 1986 to 2018.

## 2 METHODS

Our study group was created in 1986 with the co‐operation of the paediatric departments of all hospitals in Yamanashi Prefecture (10 paediatric departments in total). All children aged <15 years newly diagnosed with T1DM were registered.

Our registration system includes the following three components: (i) urinalysis conducted in all school students, (ii) information shared by study group hospitals regarding cases of new‐onset T1DM and (iii) monitoring of the numbers of patients with new‐onset T1DM who are registered to receive medical aid for ‘Specific Chronic Pediatric Diseases’. To avoid missing new‐onset cases, we asked all patients registered in the present study to register with the medical aid project.

In Japan, urinalysis is performed for all primary and junior high school students once annually in the spring under the School Health and Safety Act. The purpose of urinalysis is to screen for diseases such as nephropathies and diabetes mellitus. Students whose urine tests are positive for glucose are required to visit a clinic or hospital in Yamanashi Prefecture, and those who require hospitalization are inevitably referred to the hospitals that comprise our study group.

Every child with new‐onset T1DM is required to be referred to hospital in Yamanashi Prefecture. The major cities in Yamanashi Prefecture are located in the Kofu basin. Because of this geographical factor, children with new‐onset T1DM in the Kofu basin tend not to cross the mountains to visit medical institutions outside the Prefecture. Children with new‐onset T1DM who were under the care of private practitioners and did not receive a healthcare subsidy from the ‘Specific Chronic Pediatric Diseases’ might not have been captured in our study. Practitioners had to consult one of our study group hospitals in emergency cases as well as to meet the demands of patients’ parents, nursery staff or school teachers. Our study group members did not receive such consults from private practitioners. In Japan, hospital emergency departments are administered at the prefecture level, and ambulances do not cross prefectures. Therefore, on the basis of geographical factors, medical need and the nature of the Japanese emergency medicine system, we believe that all children newly diagnosed with T1DM would have visited the hospitals comprising our study group. Information sheets for children newly diagnosed with T1DM were collected from study group hospitals every month and used for registration. The information sheet included data on age, sex, fasting and casual plasma glucose levels, serum and urinary C‐peptide levels, haemoglobin A1c levels and serum T1DM‐related autoantibodies. We compared the number of patients with new‐onset T1DM receiving a healthcare subsidy for ‘Specific Chronic Pediatric Diseases’ in Yamanashi Prefecture with the number of study registrations to identify any omissions in each month. Because of the configuration of the registration system, the case inclusion rate was nearly 100%.

All T1DM cases prior to 2012 were diagnosed by paediatricians in each hospital. The patients were insulin‐deficient, as shown by fasting serum C‐peptide levels <0.5 ng/mL or urinary C‐peptide levels <20 μg/day. If possible, the presence of serum T1DM‐related autoantibodies [glutamic acid decarboxylase (GAD) antibody, insulin autoantibody (IAA), islet antigen‐2 (IA‐2) antibody or islet cell antibody] was evaluated. All cases after 2012 met the Japan Diabetes Society diagnostic criteria for acute‐onset T1DM. Acute‐onset T1DM was diagnosed as follows: (i) affected individuals presented with thirst, polydipsia and polyuria, leading to ketoacidosis within approximately 3 months of disease onset; (ii) affected individuals required continuous insulin therapy from early after diagnosis and may experience a transient ‘honeymoon phase’; (iii) affected individuals were confirmed positive for either GAD antibodies, IA‐2 antibodies, IAA or ZnT8 antibodies (IAA positivity must be confirmed prior to initiation of insulin therapy; and (iv) affected individuals who were not positive for IA‐2 antibodies had fasting serum C‐peptide levels <0.6 ng/mL, thus suggesting a deficit in endogenous insulin secretion. Individuals who met criteria 1‐3 were diagnosed with acute‐onset (autoimmune) T1DM (Type 1A). Those who met criteria 1, 2 and 4 were diagnosed with acute‐onset T1DM (Type 1B). Those who met criteria 1 and 2 but not 3 and 4 were re‐evaluated after a time interval with diagnosis put on hold. Those who met the criteria for fulminant T1DM were diagnosed as such. Patients who had previously developed T1DM in another prefecture and moved to Yamanashi Prefecture were not included. The mean population of Yamanashi Prefecture was approximately 850 000 people between 1986 and 2018; considering the previously measured incidence of childhood T1DM in Japan, the size of this population was not large enough to expect that new cases would be diagnosed every year. Therefore, to permit statistical accurate statistical analysis, 3‐year time bands were used.

We calculated the annual and 3‐year crude incidences of T1DM using cases registered prospectively between January 1986 and December 2018. The annual crude incidence was calculated by dividing the annual number of newly diagnosed cases by the annual population sizes of the relevant age group (0‐4 years, 5‐9 years, 10‐14 years and 0‐14 years) in Yamanashi Prefecture, as obtained from National Census data in Japan (Statistics Bureau of Japan; Home > Statistics > opulation Estimates > Result of Population Estimates; www.stat.go.jp/english/data/jinsui/2.html). The 3‐year intervals were as follows: 1986‐1988, 1989‐1991, 1992‐1994, 1995‐1997, 1998‐2000, 2001‐2003, 2004‐2006, 2007‐2009, 2010‐2012, 2013‐2015 and 2016‐2018. The 3‐year crude incidences were calculated by dividing the number of newly diagnosed cases during each period by the populations of the relevant age groups (0‐4 years, 5‐9 years, 10‐14 years and 0‐14 years) during the same 3‐year period. Both incidences were presented in raw form and following stratification by sex, age and age group (0‐4 years, 5‐9 years and 10‐14 years). The 3‐year standardized incidence among 0‐ to 14‐year‐olds was calculated by dividing the sum of the incidences among the three age groups, each multiplied by the appropriate standard population, by the sum of the standard populations. We used Segi's world standard population to calculate 3‐year standardized incidences among 0‐ to 14‐year‐olds as follows:

3‐year standardized incidence among 0‐ to 14‐year‐olds = $\frac{\sum_{i=1}^{3}N_{i}p_{i}}{\sum_{i=1}^{3}N_{i}}$,

where *N_i_* = Segi's world standard population, *N_1_* = the number of children aged 0‐4 years, *N_2_* = the number of children aged 5‐9 years, *N_3_* = the number of children aged 10‐14 years*, p_i_* = the 3‐year crude incidence in each 3‐year period, *p_1_* = the incidence among 0‐ to 4‐year‐olds, *p_2_* = the incidence among 5‐ to 9‐year‐olds, and *p_3_* = the incidence among 10‐ to 14‐year‐olds.

Poisson regression analysis was used to investigate incidence trends and to compare the results of our study with previous studies conducted in Japan and elsewhere. Statistical analyses were performed using SAS software (version 9.2; SAS Institute). Values of *p* < .05 were considered statistically significant. We calculated 95% confidence intervals (CIs) of the Poisson regression coefficients.

The study was approved by the Institutional Review Board of the School of Medicine of Yamanashi University (IRB number: 2020‐2215). We applied the opt‐out method to obtain informed consent for the analysis of anonymized patient data in this study using posters displayed in the study group hospitals. All procedures were performed in accordance with the principles laid out in the Declaration of Helsinki.

## 3 RESULTS

**Figure 1:**
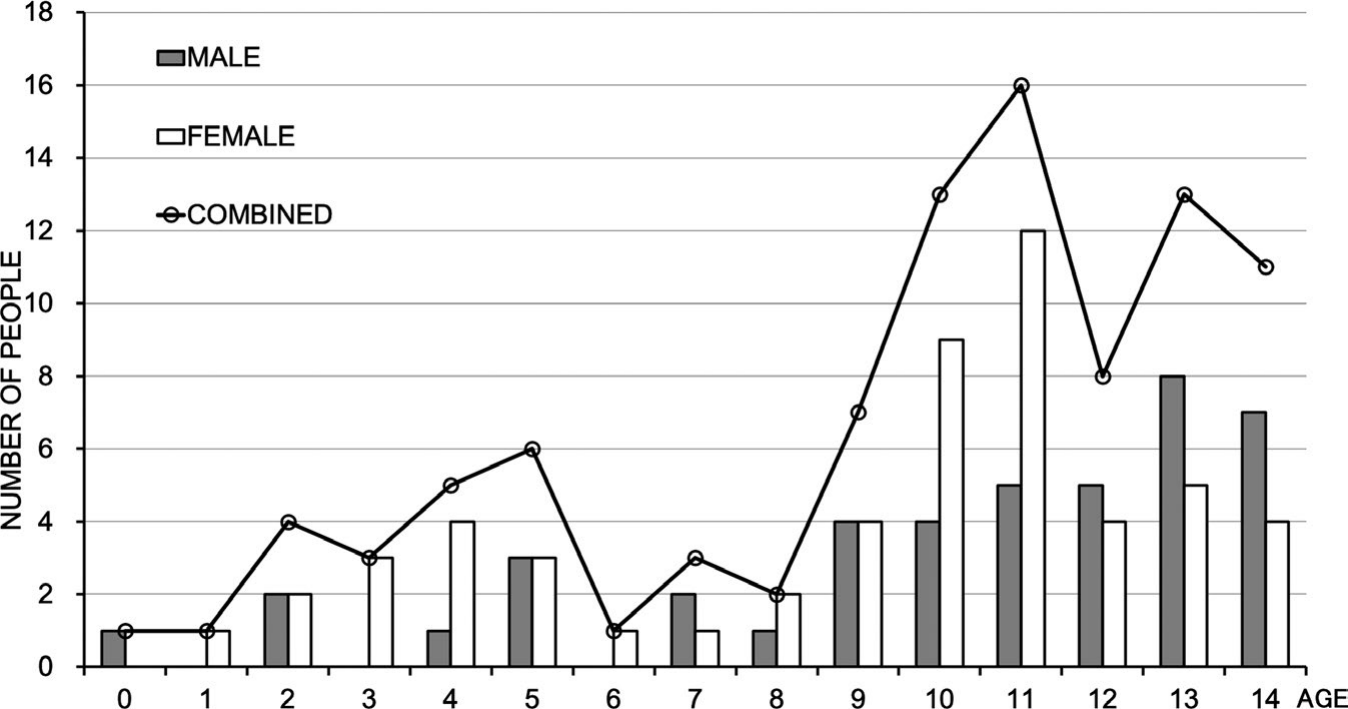
Age and sex distributions of children with type 1 diabetes in Yamanashi Prefecture between 1986 and 2018

The number of patients with new‐onset T1DM receiving a healthcare subsidy for ‘Specific Chronic Pediatric Diseases’ in Yamanashi Prefecture and the number of study registrations were identical. The total number of T1DM cases was 99 (44 boys and 55 girls; female/male ratio 1.25) over the study period. The age and sex distributions of children with T1DM were bimodal. There was an initial minor peak at age 4‐5 years followed by a second larger peak at age 10‐12 years (Figure 1). The numbers of cases and incidences for each age group during each 3‐year period are shown in Table 1.

**Table 1:** Numbers of type 1 diabetes mellitus cases and incidences (per 100 000 person‐years) among children of each age group during 3‐year periods from 1986 to 2018

	Year Period	1986–1988	1989–1991	1992–1994	1995–1997	1998–2000	2001–2003	2004–2006	2007–2009	2010–2012	2013–2015	2016–2018
0–4 years	Number at risk	147 000	139 000	135 000	135 000	132 000	125 000	115 000	107 000	101 000	94 000	89 000
	Number of cases	1	2	0	3	2	2	1	2	1	0	0
	Crude incidence	0.68	1.44	0.00	2.22	1.52	1.60	0.87	1.87	0.99	0.00	0.00
5–9 years	Number at risk	162 000	155 000	150 000	143 000	138 000	136 000	129 000	120 000	112 000	103 000	99 000
	Number of cases	1	0	2	4	1	0	2	3	3	4	2
	Crude incidence	0.62	0.00	1.33	2.80	0.72	0.00	1.55	2.50	2.68	3.88	2.02
10–14 years	Number at risk	191 000	174 000	160 000	157 000	148 000	140 000	136 000	132 000	125 000	117 000	109 000
	Number of cases	3	6	10	5	5	6	8	10	1	8	1
	Crude incidence	1.57	3.45	6.25	3.18	3.38	4.29	5.88	7.58	0.80	6.84	0.92
0–14 years	Number at risk	500 000	468 000	445 000	435 000	418 000	401 000	380 000	359 000	338 000	314 000	297 000
	Number of cases	5	8	12	12	8	8	11	15	5	12	3
	Crude incidence	1.00	1.71	2.70	2.76	1.91	1.75	2.89	4.18	1.48	3.82	1.01
	Standardized incidence	0.92	1.56	2.24	2.69	1.80	1.86	2.54	3.73	1.48	3.24	0.92

The annual incidence among 5‐ to 9‐year‐olds significantly increased between 1986 and 2018 (average annual increase 5.35%, 95% CI 2.34‐8.35%, *p* = .0005). There was a trend towards increased annual incidence among 0‐ to 14‐year‐olds between 1986 and 2018, although this was not statistically significant (average annual increase 1.16%, *p* = .3396). The annual incidences among 0‐ to 4‐year‐olds (*p* = .3532) and 10‐ to 14‐year‐olds (*p* = .7920) did not change over the study period.

**Figure 2:**
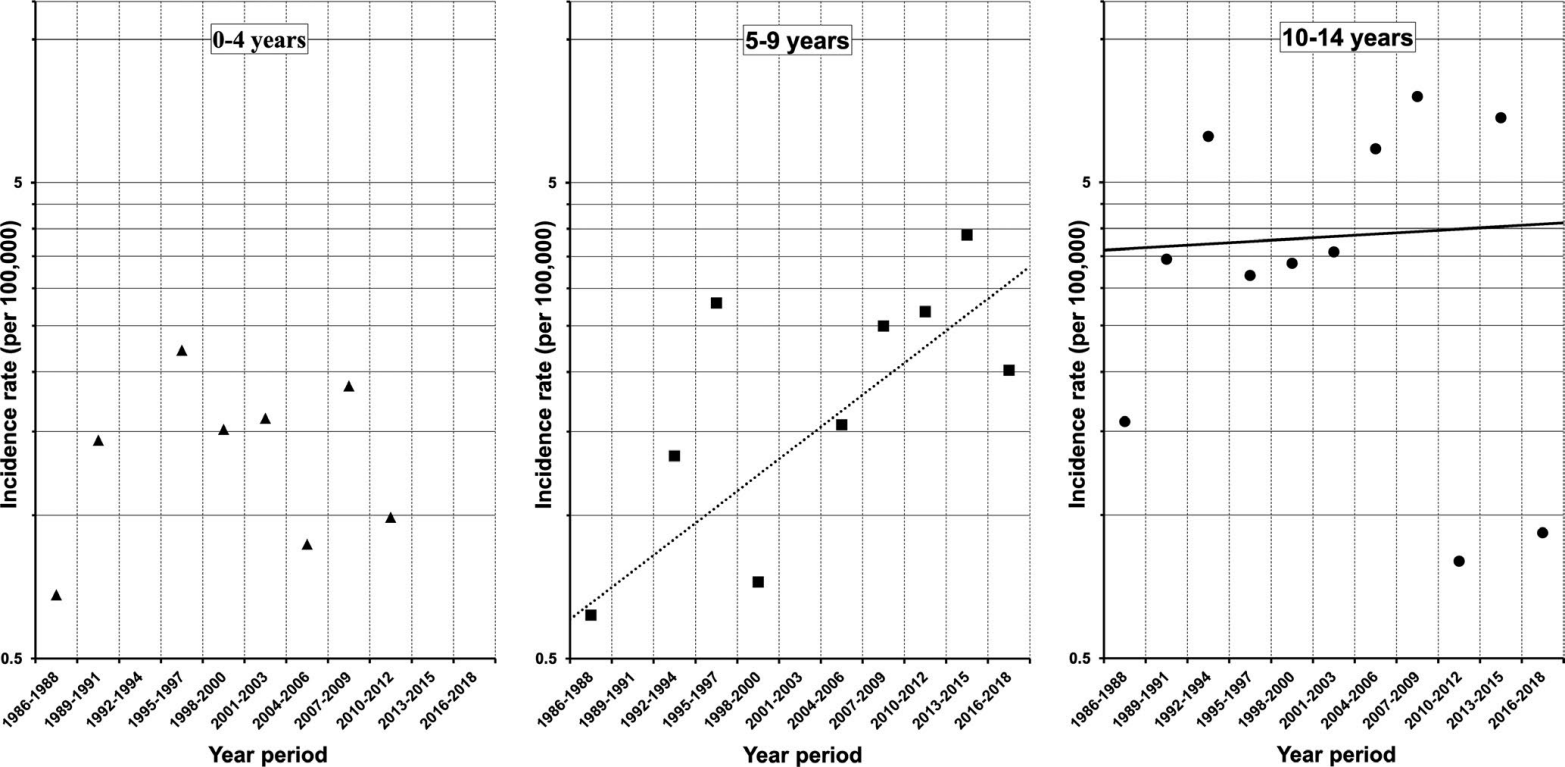
Three‐year incidences of type 1 diabetes among children of each age group in Yamanashi Prefecture between 1986 and 2018. The average increase in incidence per 3‐year interval among 5‐ to 9‐year‐old children was 15.52% (*p* = .0516, *n* = 22, Poisson regression test)

**Figure 3:**
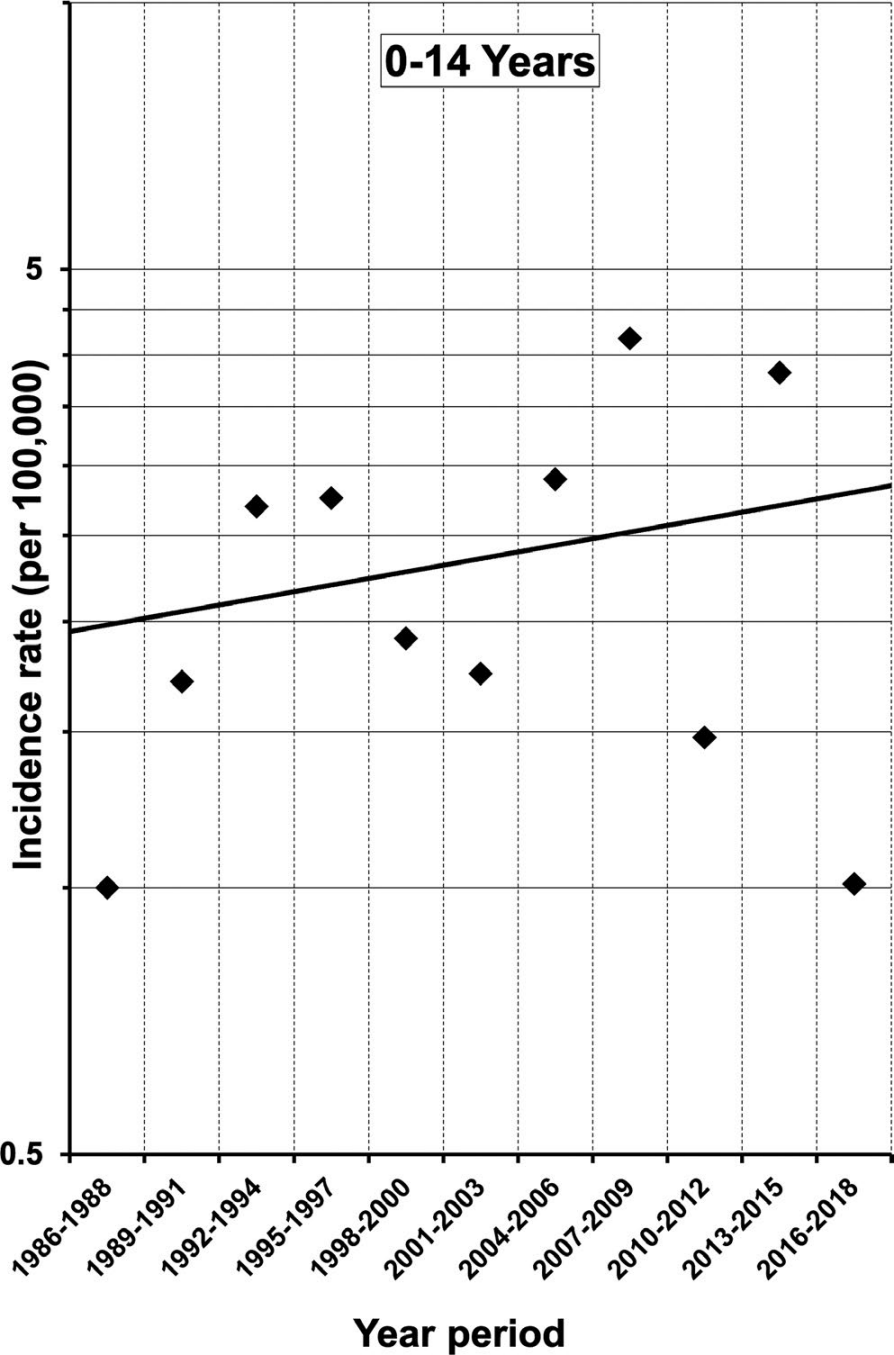
Three‐year incidences of type 1 diabetes among 0‐ to 14‐year‐old children in Yamanashi Prefecture between 1986 and 2018. The average increase in incidence per 3‐year interval was 3.45% (*p* = .5856, *n* = 98, Poisson regression test)

The 3‐year incidence of T1DM among 5‐ to 9‐year‐olds also showed an increasing trend between 1986 and 2018 (average 3‐year increase 15.52%, *p* = .0516; Figure 2), as did incidence among 0‐ to 14‐year‐olds (average 3‐year increase 3.45%, *p* = .5856; Figure 3). There were no changes in the 3‐year incidences among 0‐ to 4‐year‐olds (*p* = .4781) or 10‐ to 14‐year‐olds (*p* = .8019; Figure 2). When incidences were stratified by sex, no changes were identified over the study period.

## 4 DISCUSSION

The age and sex distributions of children with T1DM in Yamanashi Prefecture between 1986 and 2018 did not differ from those revealed by a previous study conducted in Hokkaido, Japan, between 1973 and 1992.^1^ Furthermore, the female/male ratio in our study (1.25) was similar to those reported in studies conducted in Japan between 1986 and 1990 (1.50)^11^ and in Korea between 1995‐2000 and 2012‐2014 (1.56 and 1.25, respectively).^9^ Thus, the slight sex predisposition of childhood T1DM cases in Japan appears to be shared with other countries with low incidences of childhood T1DM, such as Korea, but is distinct from the situation in countries with high incidences such as Finland. The cause of this sex difference remains unclear.^9^, ^11^, ^12^ We speculate that this difference may be attributable to Asia‐specific environmental factors such as diet, lifestyle and climate.

The annual and 3‐year incidence data for each age group and for all children aged ≤14 years observed in the present study suggest that there had been a gradual increase in T1DM incidence in children, with the largest increase occurring among 5‐ to 9‐year‐olds. The increase in incidence among children aged 0‐14 years was not statistically significant because the age group with the highest incidence (10‐ to 14‐year‐olds) showed a relatively small increase; the numbers of cases among 5‐ to 9‐year‐olds showed a greater increase but were still too small to significantly impact overall incidence.

In contrast to the present findings, Onda et al reported that the incidence of T1DM among 5‐ to 9‐year‐old Japanese children did not increase between 1998 and 2001 (2.24/100 000 person‐years) and 2005‐2010 (2.27/100 000 person‐years).^3^ However, the registration system for medical subsidies of patients with ‘Specific Chronic Pediatric Diseases’, which Onda et al analysed, may omit young newly diagnosed patients. This is because the medical aid system requires a payment for registration and for medical expenses to be initially paid up‐front. Depending on the income of the guardians, these costs can vary from ¥2000 to ¥4000.

Another medical subsidy programme is provided for all infants and children by local governments in Japan. This programme varies regionally by name, age of eligibility and the proportion of medical expenses that must be defrayed by guardians. Thanks to this municipal medical subsidy programme, the medical expenses of children aged <12 years in Japan are minimal (from zero to several hundred yen; in some regions, subsidies are available for children up to 18 years of age) and there are no costs to register. This municipal medical subsidy programme was initiated during the 2000s in parts of Tokyo for children aged <6 years and then was expanded to cover areas outside Tokyo as well as a wider age range. Thus, for the last 15 years in Japan, patients with T1DM under 12 years of age (up to 15 years of age in some regions) can receive medical care almost free of charge without applying for the medical subsidy for patients with ‘Specific Chronic Pediatric Diseases’. Therefore, it is probable that the guardians of a considerable number of children with T1DM do not register with the national medical aid project for ‘Specific Chronic Pediatric Diseases’, especially parents of young children who have low incomes. It follows that the number of recipients of healthcare subsidies for ‘Specific Chronic Pediatric Diseases’ does not accurately reflect the number of children who are newly diagnosed with T1DM, especially in the 5‐ to 9‐year‐old age group. This may be the principal cause of the discrepancy between our results and those of Onda et al.

The trends observed in our age group data were slightly different from those in Europe. In Europe, areas with lower incidence showed higher increases in T1DM incidence among 0‐ to 4‐year‐olds. Japan has one of the lowest childhood T1DM incidences in the world^4^; therefore, it would be expected that in Yamanashi Prefecture, the 0‐ to 4‐year‐old age group would show a greater increase in incidence compared with other age groups. In fact, the 5‐ to 9‐year‐old age group showed the largest rate of T1DM incidence increase in Yamanashi Prefecture. However, if the present study had been conducted in a larger population, it might have yielded similar findings to those of the EURODIAB study. The EURODIAB study group suggested that this trend might indicate a shift towards a younger age of T1DM onset in Europe,^4^ and this might also be true in Yamanashi Prefecture. A larger population must be studied to reliably detect such a trend. Wu et al^13^ reported that age group‐dependent trends in T1DM incidence in Zhejiang, China, from 2007 to 2013 were similar to those in Europe. We speculate that a shift towards younger age of onset might be occurring in Asia as well as in Europe. As the authors of these previous studies have stated, the reasons underlying these trends remain unclear.

Two long‐term data sets may provide some insight into temporal trends in the age of T1DM onset in Yamanashi Prefecture, although we caution that any connections between these data sets and T1DM age of onset are highly speculative. Yoshinaga et al analysed data from the Annual Report of the School Health Survey published by the Ministry of Education, Culture, Sports, Science and Technology, Japan, from 1978 to 2007 and reported that the prevalence of obesity among 5‐ and 8‐year‐olds had gradually decreased since the early 2000s.^14^ We reviewed additional data from the same source until 2019 and found that the prevalence of obesity among 5‐ to 9‐year‐olds continued to gradually decrease over this period (e‐Stat; Portal Site of Official Statistics of Japan; Home > Search > Japanese (Japanese page only)> Statics code; 00400002). Changes in obesity prevalence may or may not be related to trends in T1DM onset in Yamanashi Prefecture. Another population‐based long‐term data set available for analysis was the number of newborns according to birth weight (<1.0 kg, 1.0‐1.4 kg, 1.5‐1.9 kg, 2.0‐2.4 kg, 2.5‐2.9 kg, 3.0‐3.4 kg and 3.5‐3.9 kg) from 1988 to 2018 in Yamanashi Prefecture (Yamanashi Prefecture Homepage; https://www.pref.yamanashi.jp/imuka/30doutai.html; Excel file No. 13). Analysis of this data set showed that the number of newborns with birth weights >3.0 kg continuously decreased while the number of newborns with birth weights <1.9 kg remained stable from 1988 to 2018 in Yamanashi Prefecture. The TEDDY study reported that birth weight was positively associated with risk of islet autoimmunity (IA),^15^ while the Australian Baby Diab Study reported that birth weight z score was not associated with risk of IA.^16^ Changes in birth weight distribution may or may not be related to trends in T1DM onset in Yamanashi Prefecture. The TEDDY study reported that a slower rate of height increase during infancy and a faster height increase rate in early childhood were associated with progression from IA to T1DM.^15^ Unfortunately, growth data for the patients in our study were not available and there are no population‐based, long‐term surveys of height and weight increase during infancy and early childhood in Japan. Environmental factors, such as lifestyle and eating habits, might affect growth during infancy and early childhood as well as the incidence of T1DM among 5‐ to 9‐year‐olds in Yamanashi Prefecture.

Our study had several limitations. First, the number of T1DM cases diagnosed was small and a small group of T1DM patients might not have been captured for various reasons. For example, patients with new‐onset T1DM living in Yamanashi Prefecture who moved to another prefecture soon after onset, and patients with T1DM under the care of private practitioners who did not receive a healthcare subsidy from the ‘Specific Chronic Pediatric Diseases’, may not have consulted one of our study group hospitals. Failure to capture all T1DM cases would result in underestimation of the true incidence, but this potential bias probably had minor effects on our results. We believe the patients captured in our study represented almost all newly diagnosed cases of childhood T1DM registered over a 33‐year period in Yamanashi Prefecture. The trends observed in the present study were very similar to those identified in previous studies conducted in Japan and Korea.^1^, ^2^, ^9^ Collectively, these findings suggest that the incidences of T1DM among 5‐ to 9‐year‐olds and among children as a whole might be increasing in Yamanashi Prefecture and that the age of onset may be gradually decreasing, as it is in Europe.

In Japan, as in other countries, it is important to conduct surveillance for chronic diseases such as T1DM in childhood. These data provide a complete picture of chronic disease in childhood and are an important basis for the government to improve medical and welfare support systems for children.

## 5 ETHICS STATEMENT

The study was approved by the Institutional Review Board of the School of Medicine of Yamanashi University (IRB number: 2020‐2215).

## PATIENT CONSENT STATEMENT

All study participants provided informed consent obtained in the form of opt‐out.

## CONFLICT OF INTERESTS

The authors declare no conflicts of interest associated with this manuscript.

## AUTHOR CONTRIBUTIONS

Tomohi. S., Ko. K. and SA designed the study. Tomohi. S., Ko. K., Ki. K., MM, Hide. Y., KM, H.N, DW, YM, KS, Tomoa. S. and MO collected clinical data. Hiro. Y. performed statistical analysis. Tomohi. S. wrote the manuscript. Ko. K., Ki. K., MM and SA provided clinical advice. All authors read and approved the final manuscript.

## Data Availability

The data that support the findings of this study are available from the corresponding author, Saito, upon reasonable request.
